# TetR Family Regulator *brpT* Modulates Biofilm Formation in *Streptococcus sanguinis*

**DOI:** 10.1371/journal.pone.0169301

**Published:** 2017-01-03

**Authors:** Jinlin Liu, Victoria N. Stone, Xiuchun Ge, Madison Tang, Fadi Elrami, Ping Xu

**Affiliations:** 1 Philips Institute for Oral Health Research, Virginia Commonwealth University, Richmond, Virginia, United States of America; 2 Department of Microbiology and Immunology, Virginia Commonwealth University, Richmond, Virginia, United States of America; 3 Center for the Study of Biological Complexity of Virginia Commonwealth University, Richmond, Virginia, United States of America; University of Florida, UNITED STATES

## Abstract

Biofilms are a key component in bacterial communities providing protection and contributing to infectious diseases. However, mechanisms involved in *S*. *sanguinis* biofilm formation have not been clearly elucidated. Here, we report the identification of a novel *S*. *sanguinis* TetR repressor, *brpT* (Biofilm Regulatory Protein TetR), involved in biofilm formation. Deletion of *brpT* resulted in a significant increase in biofilm formation. Interestingly, the mutant accumulated more water soluble and water insoluble glucans in its biofilm compared to the wild-type and the complemented mutant. The *brpT* mutation led to an altered biofilm morphology and structure exhibiting a rougher appearance, uneven distribution with more filaments bound to the chains. RNA-sequencing revealed that *gtfP*, the only glucosyltransferase present in *S*. *sanguinis*, was significantly up-regulated. In agreement with these findings, we independently observed that deletion of *gtfP* in *S*. *sanguinis* led to reduced biofilm and low levels of water soluble and insoluble glucans. These results suggest that *brpT* is involved in the regulation of the *gtfP*-mediated exopolysaccharide synthesis and controls *S*. *sanguinis* biofilm formation. The deletion of *brpT* may have a potential therapeutic application in regulating *S*. *sanguinis* colonization in the oral cavity and the prevention of dental caries.

## Introduction

Biofilms represent a major health concern as they are ubiquitous in nature and frequently attach to biotic and abiotic surfaces [1]. Bacteria within these communities create an extracellular matrix composed of exopolysaccharides (EPS), proteins, lipids, DNA and ions [2, 3]. Attachment is first established through the adherence of bacteria to the surface through the interaction of membrane associated proteins, followed by the accumulation of bacteria and the matrix to form multi-layered clusters. This provides the bacteria with protection from external stresses, decreases susceptibility to antimicrobial therapy and immune clearance [4]. Not surprisingly, biofilm-related diseases are a cause of persistent infections, are a significant risk factor in medical-device related infections [5, 6] and are estimated to account for 80% of all bacteria-related infections [6]. Therefore, studies aimed at elucidating the mechanisms by which bacteria regulate biofilm formation are essential.

*Streptococcus sanguinis*, a Gram-positive facultative anaerobe, is a commensal microbe within the human oral cavity and is known to be a pioneering contributor to dental plaque biofilm [7–9]. The formation of dental plaque biofilm is highly organized and composed of multispecies microorganisms [10]. However, attachment of pioneering bacteria, such as *S*. *sanguinis*, to the salivary glycoprotein-coated surface is essential for the initiation of the biofilm development, as they can modify the environment to make it less harmful, aiding in the attachment of succeeding organisms [11]. While several studies have been undertaken to identify potential contributors to the biofilm-forming ability of *S*. *sanguinis* [12], the process is exceedingly complex and more are needed to better understand its roles in dental plaque and oral health.

TetR is a transcriptional regulator of the *tet* genes that encode proteins required for tetracycline resistance [13] Yet, studies show that TetR family proteins also regulate genes whose products are involved in diverse biological processes, such as multidrug resistance, biogenesis of antibiotics, osmotic stress, pathogenicity and biofilm formation [13, 14]. The TetR regulator *icaR*, in *Staphylococus aureus* is a negative regulator of intracellular adhesion genes within the *ica* operon, influencing the synthesis of polysaccharide poly-N-acetylglucosamine and biofilm formation [15, 16]. In *Streptococcus pneumoniae*, the TetR family regulator, SczA, aids in resistance against metal ions [17]. However, diverse regulation by TetR has not been observed in *S*. *sanguinis*. By screening our comprehensive *S*. *sanguinis* mutant library for changes in biofilm development [18], we identified a novel TetR repressor, *brpT* (Biofilm Regulatory Protein TetR; SSA_0144) that was shown to be biofilm-related. An amino acid sequence alignment suggests that *brpT* homologs are widely distributed amongst pathogenic or opportunistic oral streptococci (S1 Fig). However, understanding of this group of TetR family regulators in biofilm formation and other biological processes is rarely mentioned, except in *S*. *mutans* SMU.1349, which was characterized to modulate the transcription of itself and several other genes in the genomic island, TnSmu2 [19]. In this study, we show that *brpT* influences biofilm formation in *S*. *sanguinis*, deletion of *brpT* alters the spatial structure of the biofilm and increases the ability of *S*. *sanguinis* to accumulate glucans. Additionally, we perform RNA-sequencing to examine possible genes regulated by *brpT*, providing a gene expression profile for future studies.

## Materials and Methods

### Strains and growth conditions

The bacterial strains, plasmid and primers used in this study are listed in Table 1. *S*. *sanguinis* strain SK36 [20] and its derivatives were cultured in brain heart infusion (BHI) broth, on BHI agar or in biofilm medium (BM) [21]. For selective growth of *S*. *sanguinis* mutants, kanamycin was used at a concentration of 500 μg/ml and for the selection of the *S*. *sanguinis* complemented mutant; erythromycin was used at a concentration of 10 μg/ml. To test glucan synthesis in *S*. *sanguinis*, BM was supplemented with 1% sucrose (w/v).

**Table 1:** Bacterial strains, plasmids, and primers used in this study.

Strain or primer	Relevant characteristics	Source
***S*. *sanguinis***		
SK36	Human plaque isolate	(20)
Δ*brpT*	Km^R^; Δ*brpT::aphA-3*	(18)
Δ*brpT* _C	Erm^R^; *brpT*^*+*^*::pSerm*	This work
Δ*gtfP*	Km^R^; Δ*gtfP::aphA-3*	(18)
Δ*brpT/* Δ*gtfP*	Km^R^ Erm^R^; Δ*brpT::aphA-3*,Δ*gtfP::pSerm*	This work
**Plasmid**		
pVA838	Shuttle vector contains erythromycin resistant cassette able to replicate in *Escherichia coli* and *Streptococcus sanguinis*	(23)
**Primers**		
0144C-F1	GGAGGAATGAATCTATGAAACAAAC, *brpT* upstream + ORF	(18)
0144C-R1	AAATAATTCTAGGAGGGAATAATGTTCAACTCAAAATTATGAAGC, *brpT* upstream + ORF	This work
0144C-F2	TGACTAACTAGGAGGATTACATGAACAAAAATATAAAATATTCT, *erm* resistance	This work
0144C-R2	CATTATTCCCTCCTAGAATTATTTCCTCCCGTTAAATAATAG	This work
0144C-F3	TCATGTAATCCTCCTAGTTAGTCATTATGATTCACTGTGAGGTGTT, *brpT* downstream	This work
0144C-R3	GTCAAACCTCCCATAAATCTTTCAG, *brpT* downstream	(18)
0613Erm-F1	GTTGAACCCTCCTGACTTTC, upstream of *gtfP*	This work
0613Erm-R1	TTTTGTTCATAAAACCTCCTTCTG, upstream of *gtfP*	This work
0613Erm-F2	GGAGGTTTTATGAACAAAA, *erm* resistance	This work
0613Erm-R2	CTATTGCTATTTCCTCCCG, *erm* resistance	This work
0613Erm-F3	CGGGAGGAAATAGCAATAG, downstream of *gtfP*	This work
0613Erm-R3	CTGACAAGACCGTCCATAAAGC, downstream of *gtfP*	This work
*gtfP-F*	AGGCGGTGAATCTTGGCAATC, *gtfP* qRT-PCR	This work
*gtfP-*R	TGTACTTGAACCGGCTGTCC, *gtfP* qRT-PCR	This work
*gyrA*-F	CTCTGGAGATGCTTCGCGAT, *gyrA* qRT-PCR.	This work
*gyrA*-R	CCATCCCAACTGCAATCCCT. *gyrA* qRT-PCR.	This work

### Mutant construction and complementation

The single gene deletion of *brpT* and *gtfP* in *S*. *sanguinis* SK36 was previously constructed [18]. Briefly, three sets of primers were used to independently PCR amplify the 1-kb sequence upstream of the gene ORF, a promoterless kanamycin cassette (*aphA-3*) to replace the ORF and the 1-kb sequence downstream of gene ORF. The three fragments were combined through a second round of PCR. The final recombinant PCR product was transformed into *S*. *sanguinis* SK36 then selected by kanamycin resistance and confirmed by PCR analysis.

For the construction of Δ*brpT/*Δ*gtfP*, three sets of primers were used to independently PCR amplify the 1-kb sequence upstream of *gtfP*, an erythromycin cassette isolated from plasmid pVA838 [22] to replace the *gtfP* ORF and the 1-kb sequence downstream of *gtfP*. The three fragments were then combined through a second round of PCR. The final recombinant PCR product was transformed into the Δ*brpT* mutant, selected by kanamycin and erythromycin resistance then confirmed by PCR analysis.

For complementation of the *brpT* mutant, a similar PCR-based method was employed [23]. Briefly, three DNA fragments were independently amplified using primer sets 0144F1/0144R1, 0144F2/0144R2 and 0144F3/0144R3, for the 1-kb sequence upstream plus the coding sequence of *brpT*, the erythromycin resistance cassette (pVA838) [22] and the 1-kb sequence downstream of *brpT*, respectively. The final recombinant PCR product containing these three fragments was generated by overlapping PCR. It was then introduced into the *brpT* mutant to replace the kanamycin resistance cassette with the *brpT* ORF and the erythromycin resistance cassette. An erythromycin resistant and kanamycin sensitive transformant was selected and confirmed by PCR analysis.

### Detection of biofilm by crystal violet (CV) staining

Overnight cultures of *S*. *sanguinis* grown in BHI broth were diluted 1:100 into fresh BM supplemented with 1% (w/v) sucrose and 100 μl was transferred to 96-well flat bottom polystyrene microtiter plates (BIOFIL, Guangzhou, China) and incubated anaerobically for 24 h at 37°C. Biofilms were quantified by crystal violet staining (CV, Sigma, St. Louis, MO) as previously described [12]. Briefly, the plate wells were gently washed with deionized water (dH_2_O), stained with 50 μl of 0.4% (w/v) crystal violet (Fisher scientific, Pittsburgh, PA) for 15 min at room temperature and then washed 3 times with dH_2_O. The biofilm stain was dissolved in 200 μl of 33% (v/v) acetic acid and then 100 μl transferred for measuring absorbance at 600 nm. For high absorbance measurements, dissolved biofilm stains were diluted and biofilm formation was calculated as the OD_600_ measurement times the dilution factor. All samples were tested at least in triplicate. Unless stated otherwise, significance of *P* <0.05 or *P* <0.01 were determined by the Student's *t*-test.

### Quantification of glucans in biofilm

Wild-type *S*. *sanguinis* and mutants were grown in BM supplemented with 1% sucrose for 24 h in 24-well plates. The following day, the supernatant was removed and biofilms were re-suspended with an equal volume (500 μl) of distilled water, transferred into 2 ml tubes and centrifuged. The precipitate was re-suspended again with 500 μl of distilled water and centrifuged. Supernatants from these two washes were mixed and used for extraction of water soluble glucans (WSG). The sediment was dissolved in the same volume of 1 N NaOH for 3 h, and centrifuged. The supernatant of this extraction was used for the quantification of alkali-soluble glucans (water insoluble glucan, WIG). These two fractions containing WSG and WIG were precipitated separately by 3 volumes of isopropanol overnight. The precipitates obtained by centrifugation were then air dried, and dissolved in 100 μl of sterile dH_2_O (WSG) or 1 N NaOH (WIG). The amount of glucans in each fraction was quantified by the phenol-sulfuric acid method as previously described [24]. Briefly, 50 μl of each sample was pipetted into 96-well plates, and 25 μl of 5% phenol was added, then 125 μl of concentrated sulfuric acid was added rapidly and mixed by pipette. The plates were allowed to stand 30 min at room temperature. The absorbance was then measured at 490 nm. H_2_O and 1 N NaOH were used as solvents for detection of WSG and WIG, respectively, as well as standard curves, which were generated by using glucose as a reference carbohydrate. The amounts of WSG and WIG were expressed as glucose equivalent in each well. To determine the efficiency of glucan accumulation, the concentration of glucans was normalized to the concentration of genomic DNA extracted using a QIAamp DNA Mini kit (Qiagen, Hilden, Germany) and quantified by a Nanodrop 2000 spectrophotometer (Thomas Scientific).

### Confocal laser scanning microscopy (CLSM) analysis of biofilm

Wild-type *S*. *sanguinis* and mutants were grown in BM supplemented with 1% sucrose in 24-well plates as described above. The following day, the wells were gently washed three times with phosphate buffer saline (PBS, pH7.4), followed by staining with 1.5 μM SYTO 9 (Invitrogen, Grand Island, NY) for 25 min. After removing the stain, the wells were gently washed by PBS. Biofilm images were visualized and collected by CLSM using a LSM 710 confocal laser scanning system (Zeiss, Thornwood, NY). SYTO 9 fluorescence was detected by excitation 488 nm, and collected with a 493- to 559-nm bandpass filter. All *z*-sections were collected at 5.8-μm intervals by using a Plan-Neofluar ×10/0.3 objective lens. ImageJ software was used for image processing.

### Scanning electronic microscopy (SEM) analysis of biofilm

Wild-type *S*. *sanguinis* and mutants were cultured overnight. The following day, cells were diluted 1:100 in BM supplemented with 1% sucrose. Biofilms were grown on small sterile polystyrene coverslips within 24-well flat-bottom plates for 24 h at 37°C under anaerobic conditions. Biofilms on the coverslips were washed once with PBS, fixed by 2% glutaraldehyde in 0.1 M sodium cacodylate buffer for 1 h. Following dehydration through a graded series of ethanol, the coverslips were air dried and sputter coated with gold. Samples were then scoped by a SEM machine (Zeiss EVO 50 XVP, Jena, Germany).

### RNA-sequencing preparation

Wild-type *S*. *sanguinis* SK36 and the *brpT* mutant were grown in BHI medium to OD_600_ ~ 0.9. Cells were collected and treated with RNAprotect bacteria reagent (Qiagen, Valencia, CA) to stabilize RNA then broken by mechanical disruption using FastPrep lysing matrix B (Qbiogene, Irvine, CA). Total RNA was treated with DNase I (Qiagen) and prepared by using RNA easy mini kits (Qiagen) according to the manufacturer’s instructions. Ribo-Zero Magnetic Kit for Bacteria (Illumina) was used to deplete ribosomal RNA from 2 μg of total RNA. NEBNext Ultra Directional RNA Library Prep Kit for Illumina (New England BioLabs) was used for the following RNA-seq library preparation according to the manufacturer’s protocol. Briefly, ribosomal-depleted RNA was fragmented followed by first-strand cDNA synthesis from random primers using ProtoScript II Reverse Transcriptase (New England BioLabs). Second strand cDNA was synthesized and purified. End repair was performed on the double-stranded cDNA and primed with the addition of 5’-phosphorylated dA-tailed ends using T4 DNA polymerase, Klenow DNA polymerase and T4 polynucleotide kinase (New England BioLabs). This was immediately followed by adaptor ligation (New England BioLabs) and purified. Samples were PCR-amplified for 12 cycles with Phusion HiFi polymerase (New England Biolabs) with paired-end primers and a randomly chosen unique barcode (Illumina). Agencourt AMPure XP Beads (Beckman Coulter) were used for all purification steps. Library sequencing was performed by the Nucleic Acids Research Facilities at Virginia Commonwealth University using Illumina HiSeq2000. The raw RNA-seq data are available in the NCBI Gene Expression Omnibus (GEO) (www.ncbi.nlm.gov/geo/query) under the accession number: GSE89964.

### Mapping and analysis of RNA-sequencing data

Reads obtained from RNA-sequencing were aligned against the *S*. *sanguinis* SK36 genome using Rockhopper v. 2.03 [25]. Analyses were run on default parameter settings to obtain expression data of *brpT* mutant compared to wild-type SK36. Significance was determined by a q-value ≤ 0.01 adjusted for a false discovery rate of 1%. Transcriptome profiles were analyzed for enriched pathways and functionally related genes using DAVID v. 6.8 Beta [26].

### Quantitative RT-PCR (qRT-PCR) analysis

qRT-PCR was performed as described previously [23]. Wild-type *S*. *sanguinis* SK36 and mutants were grown in BHI medium with or without sucrose to OD_600_ 0.9–1.0. Cells were collected and broken by mechanical disruption using FastPrep lysing matrix B (Qbiogene, Irvine, CA). Total RNA was treated with DNase I (Qiagen) and prepared by using RNA easy mini kits (Qiagen, Valencia, CA) according to the manufacturer’s instructions. First-strand cDNA synthesis was performed in a 20 μl system containing 100 ng Total RNA, 0.2 μl Random Primer (3.0 μg/μl), 1.0 μl dNTP (10 mM each dNTP), 1.0 μl 100 mM DTT, 1.0 μl RNase OUT (40 U, Invitrogen) and 1.0 μl SuperScript III reverse transcriptase (200 U, Invitrogen), 4.0 μl first-strand buffer and RNase free water to a 20 μl volume. Reactions lacking reverse transcriptase were performed in parallel as control for possible DNA contamination. First-strand cDNA from each reaction was subjected to 10-fold dilution and used in subsequent qRT-PCR. The qRT-PCR was prepared in reactions containing 5 μl 2X SYBR Green PCR Master Mix (Applied Biosystems, Foster City, CA), 0.1 μl each PCR primer (20 μM), 0.5 μl diluted first-strand cDNA and distilled water to a 10 μl volume. The reaction was performed on an ABI 7500 fast real-time PCR system. The housekeeping gene *gyrA* was used as a normalization control. The 2^-ΔΔCt^ method was employed for calculation of relative expression levels of target genes. The data were collected and statistically analyzed from triplicates.

## Results

### *S*. *sanguinis brpT* affects biofilm formation

Through the continued study of our *S*. *sanguinis* genome-wide gene mutant library [18], we identified a new biofilm-related gene, *brpT*, a TetR family transcriptional regulator. The preliminary screening indicated that the *brpT* deletion mutant displayed an increased biofilm phenotype compared to the wild-type SK36, when grown in either BM or trypticase soy broth (data not shown).

**Fig 1:**
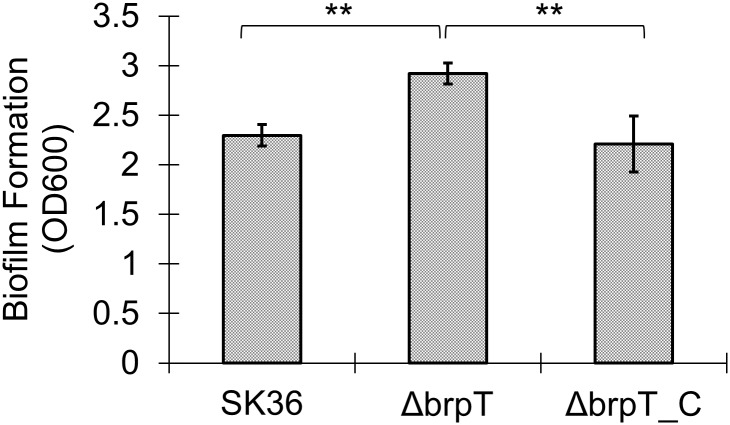
Deletion of *brpT* increases biofilm formation. *S*. *sanguinis* was cultured in BM supplemented with 1% sucrose, and biofilm biomass was determined by CV staining. Data from three biological replicates were averaged and the statistical significance between the *brpT* mutant, Δ*brpT* and the wild-type, SK36 or the complemented mutant, Δ*brpT*_C was determined by Student’s *t*-test. **, indicates significance with *P* <0.01.

To confirm that the increase in biofilm was a result of the *brpT* mutation, a complemented mutant was constructed. The growth rates of the wild-type, the *brpT* mutant and the complemented mutant were first examined and we found no significant change in the bacterial overnight growth of *brpT* mutant (S2 Fig). Biofilms for the *brpT* mutant, the wild-type and the complemented mutant were then quantified by CV staining. The *brpT* mutant showed a significant increase in biofilm formation compared to the wild-type and the complemented mutant (*P* <0.01), while no significant difference between the biofilms of the wild-type and the complemented mutant was observed (Fig 1).

BrpT is a small 22 kDa protein that contains a helix-turn-helix domain in the N-terminal region, typical of the XRE-family transcriptional regulators (Xenobiotic Response Element, a prokaryotic transcriptional DNA binding regulator family), and a tetracycline repressor domain in the C-terminal region. Previous studies indicate that the *brpT* homolog, SMU.1349, in *S*. *mutans*, is involved in the transcriptional regulation of itself and adjacent sequences [19]. To examine whether any neighboring genes were potential effector(s) responsible for the observed biofilm increase, eight mutants with deletions either upstream or downstream of *brpT* were selected from our *S*. *sanguinis* mutant library [18] and screened. We found that mutations in these neighboring genes did not significantly alter biofilm formation compared to the wild-type (S3 Fig), indicating that the observed biofilm increase was not caused by a polar effect and only the deletion of *brpT* was responsible for the observed increase in biofilm production.

### Deletion of *brpT* alters *S*. *sanguinis* biofilm properties

**Fig 2:**
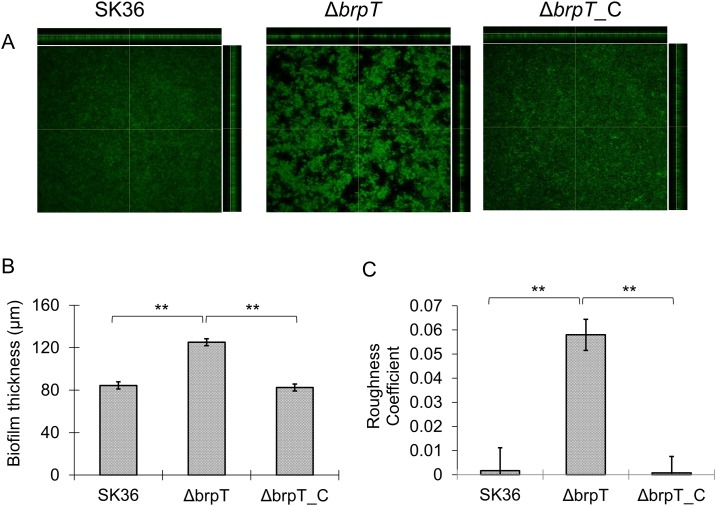
Deletion of *brpT* alters the biofilm structure. (A) Wild-type *S*. *sanguinis*, SK36, the *brpT* mutant, Δ*brpT*, and the complemented mutant, Δ*brpT*_C were grown in BM as described in *Materials and Methods*. After 24-h growth, the biofilms were washed and stained with SYTO 9, and *z*-stacks of each were acquired by CLSM with a Plan-Neofluar ×10/0.3 objective lens. Representative orthogonal views from three independent experiments are displayed. (B) Quantification of biofilm thickness by CLSM for the wild-type, Δ*brpT* and Δ*brpT*_C. (C) Quantification of biofilm roughness for the wild-type, Δ*brpT* and Δ*brpT*_C. **, indicates significance with *P* <0.01.

During our screening, we noticed that deletion of *brpT* resulted in biofilms with an uneven appearance compared to the wild-type SK36 and the complemented mutant. To better understand these morphological differences, biofilms for the wild-type, the *brpT* mutant and the complemented mutant were grown under anaerobic conditions in flat-bottom polystyrene microtiter plates and examined by confocal laser scanning microscopy (CLSM). The biofilm of the wild-type and the complemented mutant showed uniform green fluorescence intensity whereas bright fluorescence clusters surrounded by dark (blank) areas were observed for the *brpT* deletion mutant (Fig 2A). A quantitative analysis conferred with what was visually observed. The average thickness of the mutant biofilm (119.9±3.3 μm) was approximately 1.5-fold more than that of the wild-type (81.2±5.8 μm) and the complemented mutant (79.3±3.3 μm) (Fig 2B) and there was a large increase in the biofilm roughness corresponding to the uneven structural morphology (Fig 2C).

**Fig 3:**
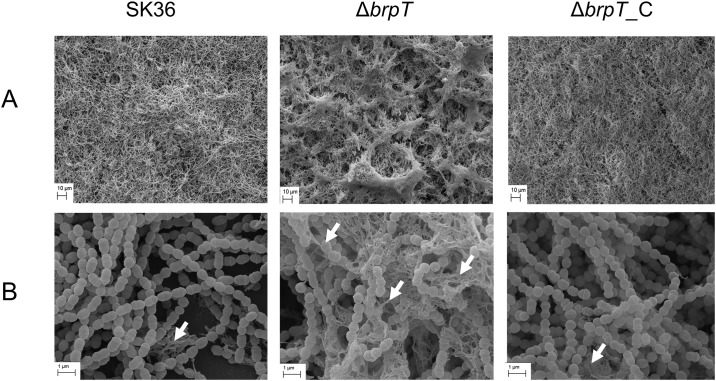
SEM analysis further reveals altered biofilm morphology and an increase in filamentous structures. Biofilms formed by the wild-type SK36, the *brpT* mutant, Δ*brpT*, and the complemented mutant, Δ*brpT_C*, scanned under (A) 1000x magnification and (B) 20,000x magnification revealed an altered morphology and an increase in filamentous structures for Δ*brpT* compared to the wild-type and complemented mutant. White arrows indicate filamentous substances.

SEM analysis was employed to further assess the changes in biofilm morphology. As shown in Fig 3A, when scanned under a low magnification (1000×), the *brpT* mutant biofilm showed a noticeably different morphology from that of the wild type. There were many peaks and dents (like a corrugated surface) compared to the relative uniform distribution of the wild-type and complemented mutant. Interestingly, the streptococcal chains of the *brpT* mutant were surrounded by numerous fine filamentous substances when scanned under a higher magnification (20,000×) (Fig 3B). These filamentous substances were also observed in SK36 and the complemented mutant, but were less abundant than that seen in the Δ*brpT* biofilms. Though further investigations should be taken towards the nature of these filaments, we hypothesize they may be water insoluble polysaccharides or glucans. These data suggest that *brpT* is involved in the regulation of the biofilm composition and structure.

### Deletion of *brpT* increases the quantity of glucans in *S*. *sanguinis* biofilm

**Fig 4:**
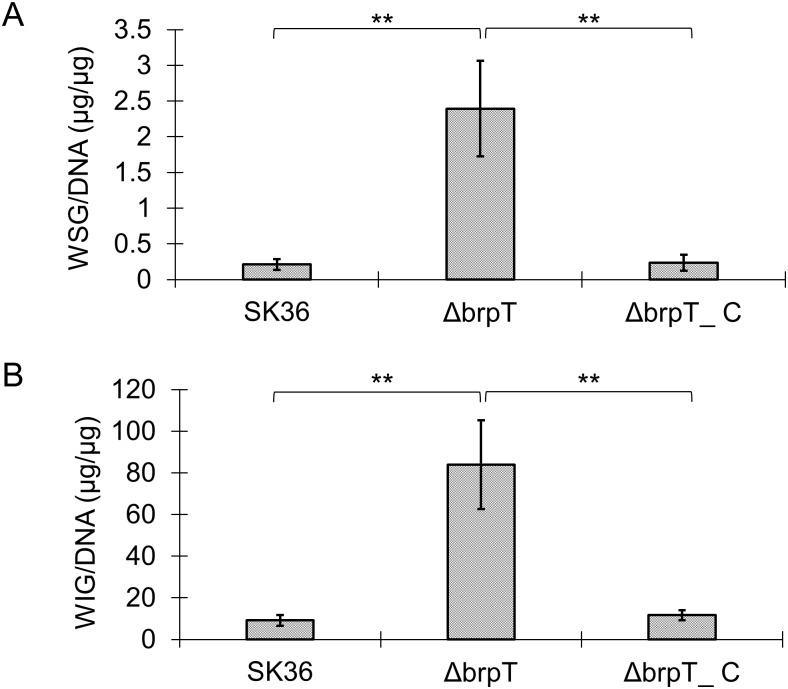
Efficiency of glucan accumulation in *S*. *sanguinis* biofilms. *S*. *sanguinis* wild-type SK36, the *brpT* mutant Δ*brpT* and the complemented mutant Δ*brpT*_C were grown anaerobically for 24 h in BM medium containing 1% sucrose at 37°C. The amounts of (A) water soluble glucans and (B) water insoluble glucans in the biofilms were quantified using the phenol-sulfuric acid method and normalized to the concentration of genomic DNA.

We noticed that the *brpT* mutant easily formed large clusters in tubes with broth supplemented with sucrose and these clusters were difficult to dissolve in water by vigorous vortex. Studies performed in other oral streptococci have demonstrated that glucans are important contributors to the bulk physical integrity and stability of biofilms and act as a key component in the matrix of oral cariogenic biofilms [27–29]. Corresponding to the increase in filamentous structures we noticed in the SEM analysis, we hypothesized that the mutant biofilm accumulated more exopolysaccharides (glucans). To examine this, static biofilms for the wild-type, *brpT* mutant and complemented mutant were scraped from polystyrene wells. Glucans were detected using a phenol-sulfuric acid method. The resulting solutions from the biofilms consisted of a water-soluble fraction for the water-soluble (WSG) glucans and an alkali-soluble fraction for the water-insoluble glucans (WIG). As shown in Table 2, *S*. *sanguinis* formed a more robust biofilm when supplied with higher sucrose concentrations and the concentration of WIG was at least 10-times more than the WSG for all of the biofilms examined. More importantly, the *brpT* mutant formed significantly more biofilm than the wild-type or the complemented with a higher accumulation of both WIG and WSG regardless of the sucrose concentration (S4 Fig). To compare the efficiency of glucan accumulation, the amount of glucans in each biofilm was normalized to the genomic DNA. As shown in Fig 4, the *brpT* mutant was more efficient at accumulating both WSG and WIG than either the wild type or the complemented mutant. These results suggest that *S*. *sanguinis* glucan synthesis is sucrose-dependent and biofilm development is influenced by *brpT* regulated glucan accumulation.

**Table 2:** Determination of glucans accumulated in *S*. *sanguinis* biofilm.

Strain	Water soluble glucans (μg/well ^a^)			Water insoluble glucans (μg/well)		
	0.25%	0.5%	1.0%	0.25%	0.5%	1.0%
SK36	0.36±0.10	0.52±0.03	1.79±0.64	4.69±0.95	14.81±2.18	77.32±21.53
Δ*brpT*	0.55±0.03 ^b^^,^ ^c^	5.70±1.41 ^d^	17.70±4.94 ^d^	23.18±7.00 ^b^^,^ ^c^	72.92±14.08 ^d^	620.83±157.22 ^d^
Δ*brpT_* C	0.30±0.06	0.54±0.13	1.72±0.82	4.21±1.23	17.06±4.78	84.64±17.56

^*a*^: 5 μl of overnight *S*. *sanguinis* cultures were added to 495 μl fresh BM in 24-well flat bottom polystyrene microtiter plates, which contained either 0.25%, 0.5% or 1% (w/v) sucrose and incubated anaerobically for 24h at 37°C. The supernatants were removed and biofilm associated glucans were determined. All samples were tested at least in triplicate. Results represented mean ± SD.

^*b*^: *P* <0.05 compared with SK36 of same culture conditions.

^*c*^: *P* <0.01 compared with *ΔbrpT*_C of same culture conditions.

^*d*^: Significance relative to SK36 or Δ*brpT*_C of same culture conditions (*P* <0.01).

### Transcriptome analysis of *brpT* regulated genes

To further investigate genes that may be influenced or regulated by *brpT*, RNA-seq was performed for a genome-wide transcriptome analysis. The *brpT* mutant and the wild-type SK36 were grown in BHI and cells were harvested at mid-log growth phase. RNA-seq data revealed 1074 genes with a significant change in expression compared to the wild-type; 594 genes were down-regulated and 480 genes up-regulated (S1 Table). Approximately 27% of the down-regulated and 29% of the up-regulated genes encoded hypothetical proteins, with a majority of those processing transmembrane domains and therefore membrane related. This was somewhat expected as membrane proteins are thought to play a crucial role in biofilm formation through cell-cell interaction, surface attachment, protein binding, nutrient transport and enzymatic activity [30]. A pathway analysis through DAVID showed a statistically significant enrichment for ribosomal proteins, translation initiation factors, tRNA synthetases and elongation factors [31]. This was followed by a significant enrichment of genes involved in fatty acid and lipid metabolism. Of the up-regulated genes there were a number of membrane associated proteins such as lipoproteins, penicillin-binding proteins, histidine transport permeases and genes for ethanolamine metabolism. We noted several multidrug ABC transport systems within the down-regulated genes. The transcriptome analysis suggested many genes are regulated by *brpT*.

**Fig 5:**
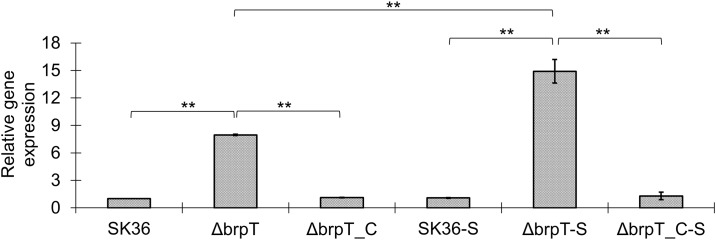
Increased gene expression of *gtfP* in the *brpT* mutant. qRT-PCR was used to determine the relative expression of glucosyltransferase, *gtfP*, in the *brpT* mutant using *gyrA* as an internal control. Data shown are mean±SD from three biological replicates. “–S” on the *x*-axis represents samples cultured in BHI supplemented with 1% sucrose. **, indicates significance with *P* <0.01.

Notably, RNA-sequencing for the *brpT* mutant showed more than a 9-fold increase in *gtfP* expression compared to the wild-type. This was verified by qRT-PCR where an 8-fold increase was observed. When BHI was supplemented with 1% sucrose and *gtfP* expression was re-analyzed by qRT-PCR, we observed a 15-fold increase in expression (Fig 5). This indicated the expression level of *gtfP* in the *brpT* mutant was significantly stimulated by sucrose (1.9-fold, *P* <0.01), whereas neither the wild-type nor the complemented mutant showed a significant increase in *gtfP* levels after sucrose stimulation. These results suggest that *brpT* has a role in repressing the expression of *gtfP* and de-repressed *gtfP* expression in the *brpT* mutant is stimulated by sucrose.

**Fig 6:**
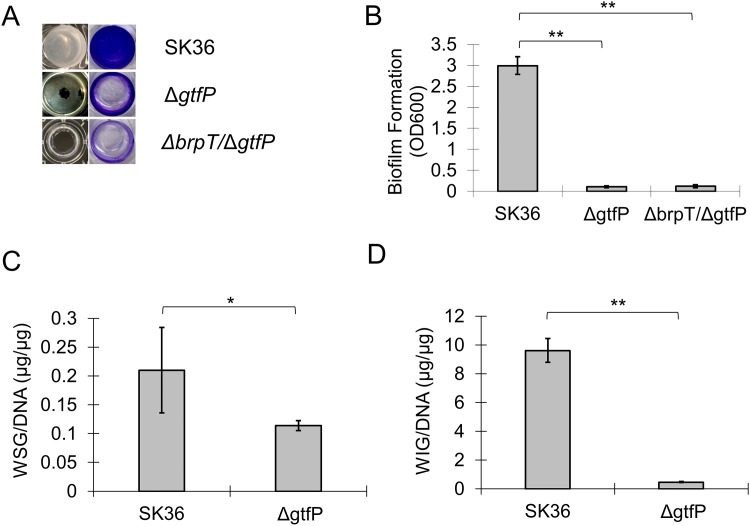
Deletion of *gtfP* in *S*. *sanguinis* decreases biofilm attachment and glucan synthesis. Wild-type, the *gtfP* mutant, Δ*gtfP*, and the double mutant, Δ*brpT*/Δ*gtfP*, were cultured in BM with 1% sucrose for 24 h anaerobically and analyzed. (A) Weak attachment of the Δ*gtfP* and the Δ*brpT*/Δ*gtfP* biofilm (pellicle) to the polystyrene surface and reduced biofilm biomass determined by CV staining. (B) Quantification of biofilm formation (OD_600_). Quantification of (C) water soluble glucans, WSG and (D) water insoluble glucans, WIG accumulated within the biofilm. **, indicates significance with *P* <0.01.

To further link the expression of glucosyltransferase to our increased biofilm phenotype in Δ*brpT*, we next examined the single deletion mutant, Δ*gtfP* [18] and the double mutant Δ*brpT*Δ*gtfP*. While both mutants could form pellicles on the bottom of the well similar to the wild type, the pellicles were loosely attached to the polystyrene surface and were easily washed away (Fig 6A). In addition, the biofilm biomass (Fig 6B) and the ability to synthesize glucans (Fig 6C and 6D) were significantly less than the wild type.

As Δ*gtfP* and Δ*brpT*/Δ*gtfP* still had the ability to form biofilm pellicles but showed reduced biomass and a significantly lost in attachment, we hypothesize that *brpT* influences the ability of the *S*. *sanguinis* biofilm to strongly adhere to surfaces through the regulation of WSG and WIG accumulation within the biofilm.

## Discussion

*S*. *sanguinis* is a pioneering colonizer within the oral cavity, initiating the establishment of disease-contributing plaque biofilm. Therefore, identifying and understanding genes that are involved in biofilm formation are critical to develop novel therapeutic strategies against oral diseases. Here, we present the first TetR family transcriptional regulator shown to influence biofilm formation in *S*. *sanguinis*. These transcriptional regulators are particularly interesting as they not only function as a repressor in controlling tetracycline resistance, but are shown to play key roles in regulating genes involved in a diverse range of adaptive responses [13, 14]. From our continued studies with the *S*. *sanguinis* genome-wide mutant library [18], we noted that deletion of the TetR gene SSA_0144, which we named *brpT*, increased biofilm formation and altered the biofilm structure. We also observed an increase in the quantity of water soluble and insoluble glucans in the biofilms. This led us to examine the glucan synthesizing enzyme, glucosyltransferase (*gtfP)*. Interestingly, RNA-seq analysis showed the expression of *gtfP* was significantly up-regulated in the *brpT* mutant and deletion of *gtfP* in *S*. *sanguinis* significantly decreased glucan biosynthesis and biofilm adhesion. This suggested that *brpT* may influence the biofilm of *S*. *sanguinis* through *gtfP*.

Bacterial exopolysaccharides are a primary component of extracellular polymeric substances (EPS) or matrices in biofilms, and their role in the development and stabilization of biofilms have been extensively studied [2, 32–34]. Sucrose obtained from the diet and utilized by oral streptococci is considered a major factor in dental caries as it is fermentable and can serve as a substrate for extracellular and intracellular polysaccharides [35, 36]. It has been noted that *S*. *mutans*, a key contributor to dental caries, utilizes three glucosyltransferases for the conversion of sucrose into polysaccharides. The glucosyltransferases, GtfB and GtfC, catalyze the synthesis of water-insoluble glucans, and GtfD, is responsible for water-soluble glucan synthesis [37, 38]. GtfP, the sole *S*. *mutans* Gtf homolog in *S*. *sanguinis*, exhibits 59%, 49% and 49% protein sequence similarity to *S*. *mutans* GtfD, GtfB and GtfC, respectively, and has been revealed to play a role in the synthesis of WSG for *S*. *sanguinis* [39]. Our RNA-seq and subsequent qRT-PCR analyses for the *brpT* mutant showed a significant increase in expression of *gtfP*, indicating a possible relationship between *brpT* and *gtfP* in exopolysaccharide production and biofilm formation. When *gtfP* was knocked out from either the wild-type or the Δ*brpT* strain it formed a weak biofilm that could only form pellicles loosely attached to a polystyrene surface (Fig 6A). While the *brpT* mutant accumulated significantly more WSG and WIG than the wild type or the complemented mutant (Table 2), deletion of *gtfP* reduced the ability of *S*. *sanguinis* to synthesize both WSG and WIG by about 2-fold and 20-fold, respectively (Fig 6C and 6D). The sole glucosyltransferase in *S*. *sanguinis* not only promotes WSG synthesis, but more importantly, determines the biogenesis of WIG in the biofilm, which may be the predominant contributor to biofilm adhesion on abiotic surfaces. This correlates with previous findings that formation of *S*. *mutans* microcolonies on saliva-coated hydroxyapatite surfaces was determined largely by the *gtfB* (associated with WIG) or *gtfC* (associated with WIG and WSG) and *gtfB* [40]. While these results indicate that *brpT* influences biofilm formation in a glucan dependent manner, mediated through the expression of *gtfP*, it is not clear whether BrpT directly or indirectly regulates *gtfP*. A bioinformatic prediction presented few known TF binding sites at 77 bp- (*argR2*), 75 bp- (*ihf*), and 36 bp- (*rpoD16*) upstream of the GtfP coding sequence but it is unclear as to whether BrpT recognizes these sites.

The contribution of exopolysaccharides to the biofilm three-dimensional architecture and modulation of inter-biofilm interactions has been documented in *S*. *mutans* [41, 42]. Here we also observed that alteration in exopolysaccharide content led to changes in the *S*. *sanguinis* biofilm structure. The *brpT* mutant produced high levels of glucans and exhibited a rough and uneven biofilm surface. As shown by the CLSM analysis, an increase in glucan synthesis led to an increase in biofilm thickness, indicating the importance of glucans in the maintenance of *S*. *sanguinis* biofilm spatial structure. Furthermore, SEM analysis gave us possible explanations for these structural changes. The bacterial chains of the *brpT* mutant biofilm were bound by numerous filaments. Since the biofilm was washed by PBS prior to fixation, we hypothesized that these filamentous substances were composed mainly of water insoluble glucans. And the wild-type *S*. *sanguinis* biofilm with normal quantities of filaments showed a relatively flat and thin biofilm. Collectively, these observations implicate *brpT* as a novel regulator of *S*. *sanguinis* biofilm structure through glucan biosynthesis.

Although many factors have been characterized to modulate the expression of Gtf and biofilm formation, the mechanisms involved in these processes still need further illustrations. In *S*. *mutans*, the expression of *gtf* depends on quorum sensing [43], carbohydrate sources, pH [44], *vicRK* [45], *frp* [46], vicX [47] as well as chemical agents [48–50]. Unlike *S*. *mutans*, *S*. *sanguinis* biofilm formation was independent of AI-2 quorum sensing [51], and *vicRK* exhibited a negative regulation of *gtfP* transcription at certain growth stages [52], making the regulation *S*. *sanguinis gtfP* more complicated. Here, our data suggest that *gtfP* in *S*. *sanguinis* is negatively regulated by the novel TetR family repressor, *brpT*. Considering that exopolysaccharides can bind to the bacterial chain and maintain the spatial structure, which is essential for the biofilm integrity, the right expression levels of exopolysaccharides might be critical for biofilm development and maturation. If exopolysaccharides on the cell surface are low, like the *gtfP* mutant, the ability to attach to an abiotic surface would be lost and biofilm development impaired. However, bacteria that produce too much exopolysaccharides might have a negative influence. This would hamper bacteria movement and biofilm diffusion, as with the *brpT* mutant biofilm, where many empty areas were observed surrounding bright green clusters (Fig 2). Were bacteria bound tightly by large amounts of sticky glucans on the cell surface, restricting biofilm diffusion? Interestingly, a similar concern was raised previously. A *P*. *aeruginosa* mutant, Δ*sadC*, produced less Pel polysaccharides and exhibited increased swarming ability, even in high-viscosity medium [53]. In light of the findings described here, the possibility should be considered that *brpT* and *gtfP* cooperate, modulating *S*. *sanguinis* biofilm formation and dispersion. Further studies may obtain more evidence to elucidate this theory.

One of the primary findings of the present study is that the GtfP-catalyzed glucan synthesis in *S*. *sanguinis* biofilm is controlled by the TetR repressor BrpT. As the BrpT homologs are widely spread in oral *Streptococci* (S1 Fig), it is possible that the role of TetR in exopolysaccharides production and biofilm formation is conserved. Given that exopolysaccharides can facilitate the expression of virulence factors in mixed-species oral biofilms [42], further investigations into the relationship between *brpT* and *gtfP* may generate new insight into oral biofilm development and provide new targets for the design of effective anti-caries therapeutics. For example, methods for increasing the expression or activity of *brpT* homologs in oral pathogens may aid in inhibiting biofilm formation and accelerate pathogen clearance.

## Supporting Information

S1 Fig Amino acid sequence alignment of BrpT and oral streptococci homologs. Amino acid residues with similarity >50% were shaded in black and >33% were shaded in gray. The Genbank aceession numbers: *S*. *sanguinis* BrpT, YP_001034156.1; *S*. *intermedius* TetR, GAD41027.1; *S*. *constellatus* TetR, WP_006270368.1; *S*. *anginosus* TetR, YP_008508598.1; *S*. *oralis* TetR, EFE56457.1; *S*. *tigurinus* TetR, EMG31875.1; *S*. *mitis* TetR, EFM31136.1; *S*. *salivarius* TetR, KEO45415.1; *S*. *sobrinus* TetR, EMP71536.1; *S*. *mutans* TetR, NP_721716.1.
(TIF)

S2 Fig Growth curves of *S*. *sanguinis* strains. Bacteria cultured overnight were diluted 1:100 into 96-well flat-bottom microplates. The OD_450_ was recorded with a microplate reader (BioTek, Thorold, Canada) every 30 min for 20 h at 37°C under aerobic conditions. The growth curves were obtained from the average of at least three repeats.
(TIF)

S3 Fig Influence of deletion in genes adjacent to *brpT* on *S*. *sanguinis* biofilm formation. Biofilm formed by wild-type *S*. *sanguinis* SK36 and single-gene deletion mutants Ssx_0140 to Ssx_0149 were tested and only the *brpT* mutant (Ssx_0144) showed a significant difference (*P* <0.01, Student’s *t*-test) relative to SK36.
(TIF)

S4 Fig *S*. *sanguinis* sucrose-dependent biofilm formation. Biofilm formed by wild-type *S*. *sanguinis* SK36, the *brpT* mutant, Δ*brpT* and the complemented mutant, Δ*brpT*_C grown in BM supplemented with either 0.25%, 0.50 or 1% sucrose. **, indicates significant difference with *P* <0.01.
(TIF)

S1 Table RNA-seq analysis of gene expressions significantly changed in the *brpT* mutant compared to the wild-type. (XLSX)
