# Supplementary figures and images for: TetR Family Regulator brpT Modulates Biofilm Formation in Streptococcus sanguinis
Source: PLoS One. 2017 Jan 3;12(1):e0169301. doi: 10.1371/journal.pone.0169301 (PMC5207742; doi:10.1371/journal.pone.0169301)

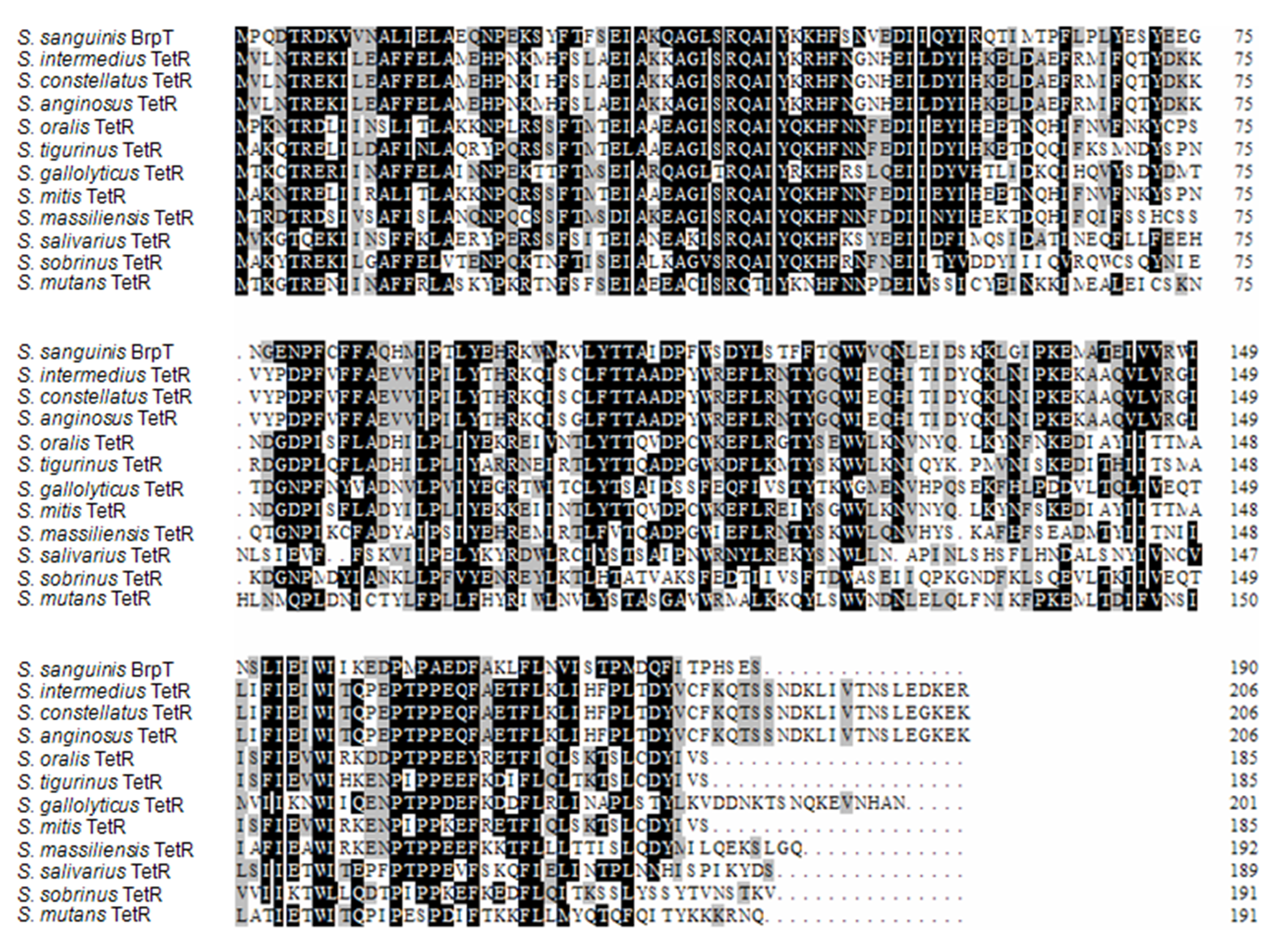

Supplement: S1 Fig — Amino acid residues with similarity >50% were shaded in black and >33% were shaded in gray. The Genbank aceession numbers: S. sanguinis BrpT, YP_001034156.1; S. intermedius TetR, GAD41027.1; S. constellatus TetR, WP_006270368.1; S. anginosus TetR, YP_008508598.1; S. oralis TetR, EFE56457.1; S. tigurinus TetR, EMG31875.1; S. mitis TetR, EFM31136.1; S. salivarius TetR, KEO45415.1; S. sobrinus TetR, EMP71536.1; S. mutans TetR, NP_721716.1. (TIF) [file pone.0169301.s001.tif]

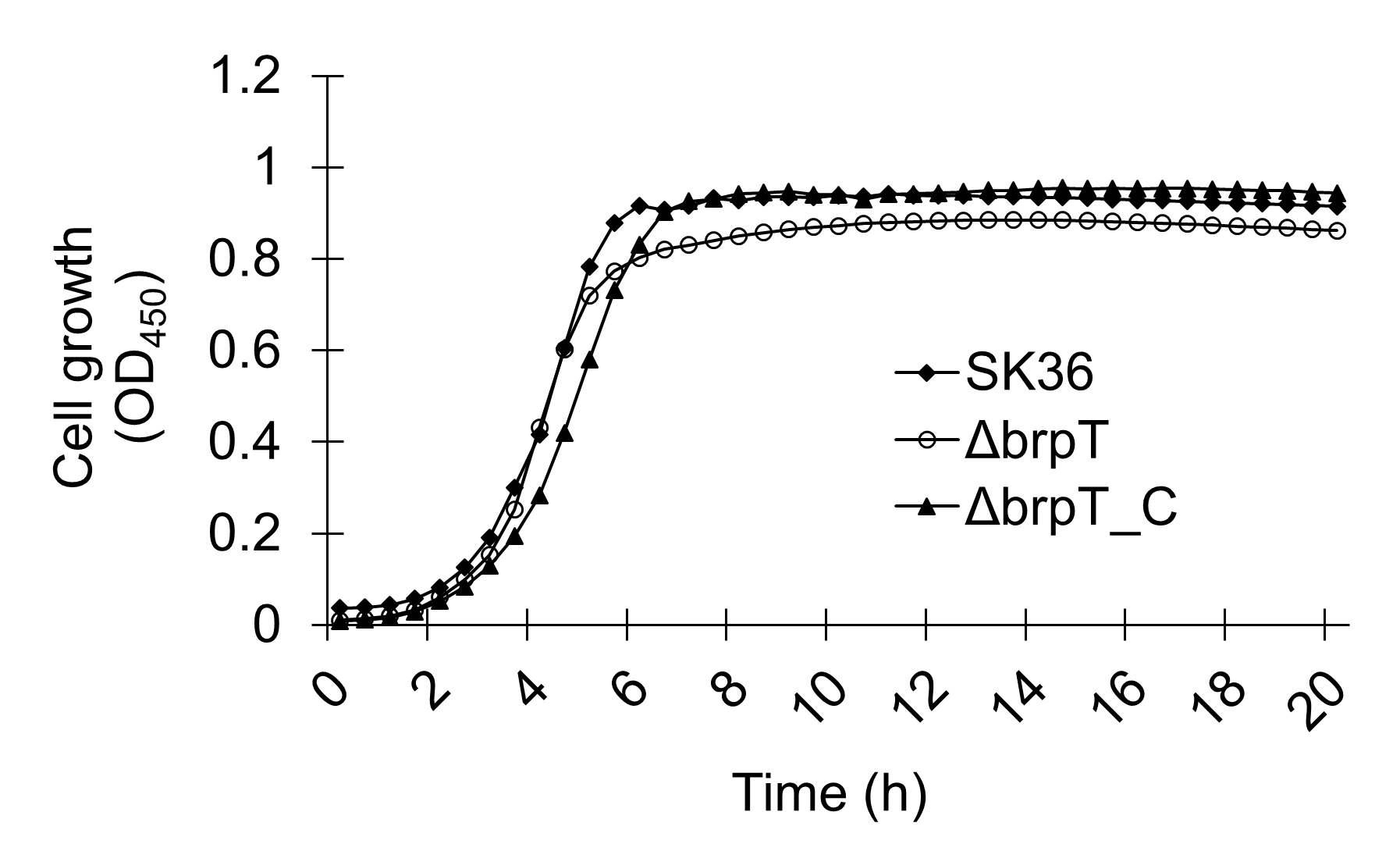

Supplement: S2 Fig — Bacteria cultured overnight were diluted 1:100 into 96-well flat-bottom microplates. The OD450 was recorded with a microplate reader (BioTek, Thorold, Canada) every 30 min for 20 h at 37°C under aerobic conditions. The growth curves were obtained from the average of at least three repeats. (TIF) [file pone.0169301.s002.tif]

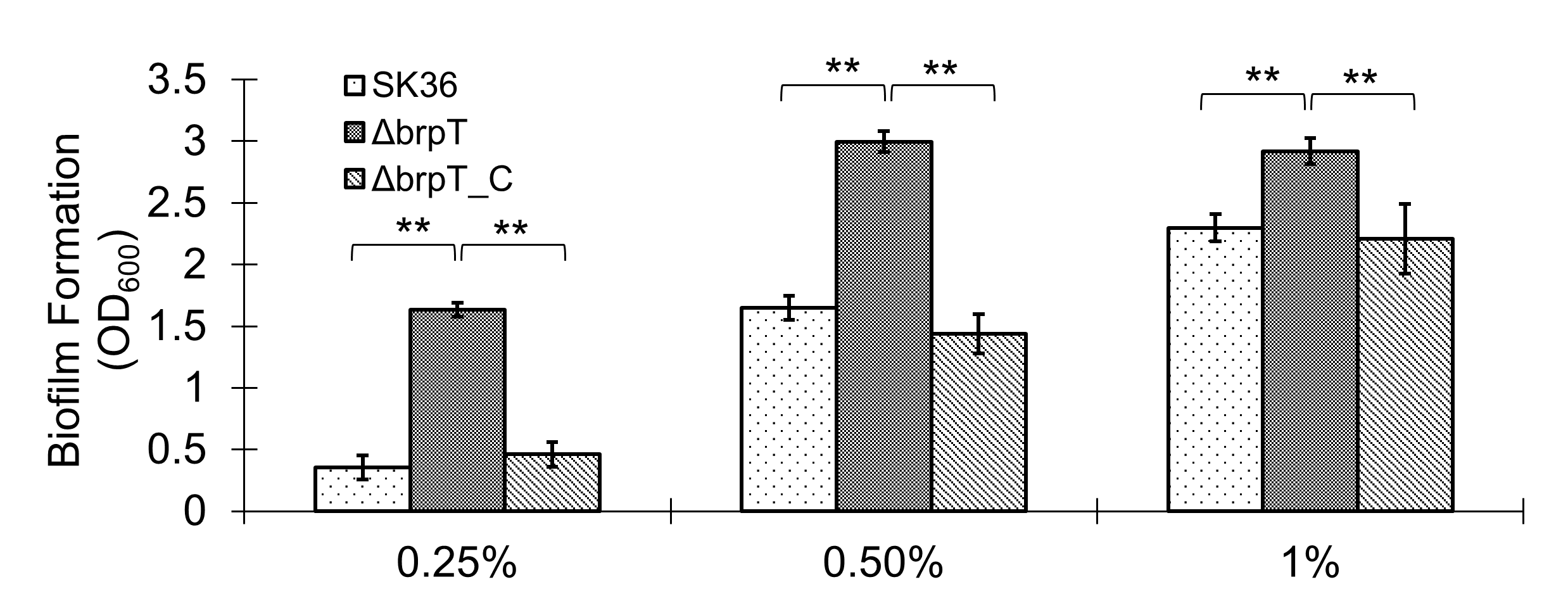

Supplement: S3 Fig — Biofilm formed by wild-type S. sanguinis SK36 and single-gene deletion mutants Ssx_0140 to Ssx_0149 were tested and only the brpT mutant (Ssx_0144) showed a significant difference (P <0.01, Student’s t-test) relative to SK36. (TIF) [file pone.0169301.s003.tif]

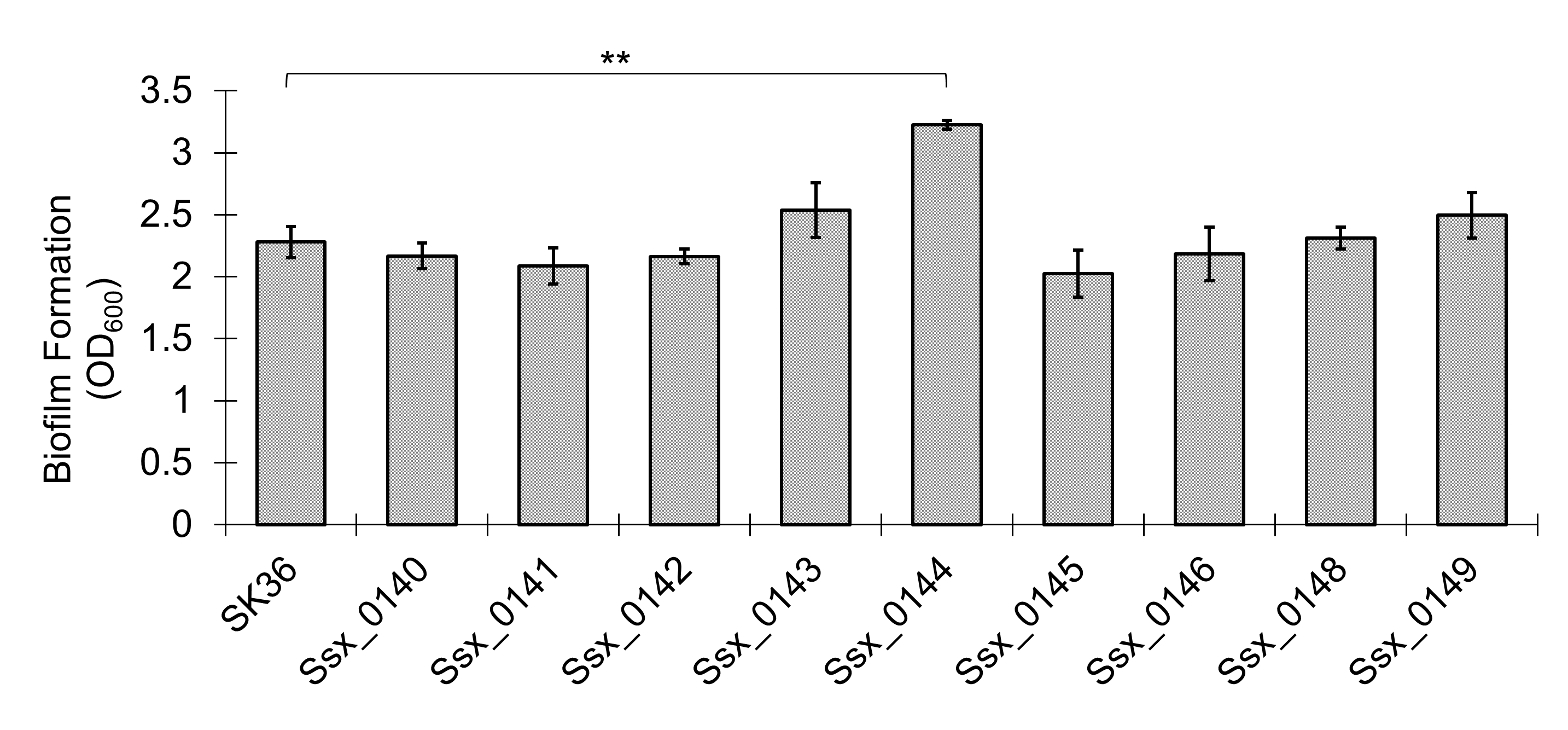

Supplement: S4 Fig — Biofilm formed by wild-type S. sanguinis SK36, the brpT mutant, ΔbrpT and the complemented mutant, ΔbrpT_C grown in BM supplemented with either 0.25%, 0.50 or 1% sucrose. **, indicates significant difference with P <0.01. (TIF) [file pone.0169301.s004.tif]
